# Integrating across memory episodes: Developmental trends

**DOI:** 10.1371/journal.pone.0215848

**Published:** 2019-04-22

**Authors:** Yee Lee Shing, Carsten Finke, Martina Hoffmann, Anna Pajkert, Hauke R. Heekeren, Christoph J. Ploner

**Affiliations:** 1 Institute of Psychology, Goethe-Universität Frankfurt, Frankfurt am Main, Germany; 2 Center for Lifespan Psychology, Max Planck Institute for Human Development, Berlin, Germany; 3 Department of Neurology, Charité-Universitätsmedizin, Berlin, Germany; 4 Department of Education and Psychology, Freie Universität, Berlin, Germany; University of Zurich, SWITZERLAND

## Abstract

Memory enables us to use information from our past experiences to guide new behaviours, calling for the need to integrate or form inference across multiple distinct episodic experiences. Here, we compared children (aged 9–10 years), adolescents (aged 12–13 years), and young adults (aged 19–25 years) on their ability to form integration across overlapping associations in memory. Participants first encoded a set of overlapping, direct AB- and BC-associations (object-face and face-object pairs) as well as non-overlapping, unique DE-associations. They were then tested on these associations and inferential AC-associations. The experiment consisted of four such encoding/retrieval cycles, each consisting of different stimuli set. For accuracy on both unique and inferential associations, young adults were found to outperform teenagers, who in turn outperformed children. However, children were particularly slower than teenagers and young adults in making judgements during inferential than during unique associations. This suggests that children may rely more on making inferences during retrieval, by first retrieving the direct associations, followed by making the inferential judgement. Furthermore, young adults showed a higher correlation between accuracy in direct (AB, BC) and inferential AC-associations than children. This suggests that, young adults relied closely on AB- and BC-associations for making AC decisions, potentially by forming integrated ABC-triplets during encoding or retrieval. Taken together, our findings suggest that there may be an age-related shift in how information is integrated across experienced episodes, namely from relying on making inferences at retrieval during middle childhood to forming integrated representations at different memory processing stages in adulthood.

## Introduction

Memory enables us to use information from our past experiences to guide new behaviours. An important part of this memory function entails the process of forming inference across multiple distinct episodic experiences [1]. For example, when seeing a woman (A) with a child (B) at the playground, then seeing the same child (B) on another day with a man (C), one may infer that the woman (A) and the man (C) are related (e.g. as partners). Evidence from the literature suggests that the hippocampus supports memory inference through novelty detection and pattern completion mechanisms, that is by detecting novelty in certain features of the newly experienced event (i.e. B), and reactivating previously stored, overlapping memory (i.e. A; [2]). At the same time, prefrontal cortex is implicated in memory inference. For example, inferior frontal gryus may be involved in making inferential judgments from premise associations during retrieval [3]. Also, medial prefrontal cortex may influence memory integration by representing relevant mental models (e.g., schema knowledge, see review by [4]).

Episodic memory improves rapidly from early childhood to adolescence [5–7]. In our own work, we demonstrated that the pace and pattern of these improvements are generally in line with the observation that medial-temporal lobe regions mature comparatively earlier than prefrontal cortex regions, which show signs of immaturity up to young adulthood and are implicated in age-related improvement in memory [7–9]. While there has been an accumulation of knowledge about the development of associative memory (i.e. memory for direct, bound events such as A–B, C–D, e.g., [10]), we know relatively little about the age gradient of memory integration.

Interestingly, in a related field on reasoning, the investigation of transitive inference has a long tradition in developmental psychology. Early studies debated about the task-situational conditions that influence children's ability to solve transitivity problems [11]. More recent research shows a revived interest in transitive inference and its development. For example, Townsend and colleagues [12] tested 6-, 8-, 10-year-olds and adults on a transitive inference task. They showed that, although children as young as age 6 were capable of learning relations between direct object pairs (i.e. AB, BC), performance on the transitive pairs (i.e. AC) showed a developmental shift later between 8 and 10 years of age.

In a recent paper, Schlichting and colleagues [13] took a direct look at memory inference comparing children (6–11 years), adolescents (12–16 years), and adults (18–30 years). Using an associative inference task, participants learned the same set of overlapping pairs of artificial objects across four study-test iterations. This meant that each object pair was learned and tested four times. Following this, participants completed an inference test, in which they had to link indirectly related objects through their common association with the corresponding overlapping object. It was found that performance on memory inference was lower than direct memory in children (with the largest difference) and adolescents, but not in young adults. This suggests that memory inference continues to improve through childhood and beyond.

In the current study, we compared children, adolescents, and young adults on their ability to form memory integration across overlapping associations. We examined memory inference using a modified version of the associative inference task [14]. Here, participants also learned a set of overlapping AB- (object-face) and BC- (face-object) associations and were then directly tested on memory of these associations (“direct” trials) and on inferential AC-associations (“inference” trials). Participants were tested with four encoding/retrieval cycles with unique pairs. In other words, each pair was used only in one cycle and the need to make memory inference decision was instructed clearly from beginning on. The formation of the integrative representation can be achieved either at encoding of BC (termed integrative encoding), or at retrieval (termed retrieval inference; [2]). As shown in Pajkert et al. [14], across cycle, healthy middle-age participants showed an increasing reliance on integratively encoded representations for AC-decisions. That is, with repeated necessity of AC-decisions, participants might have increasingly tended to form integrated ABC-representations when encoding the BC-pairs (by reactivating the corresponding AB-pair). Unlike Schlichting et al. [13] who testing for memory inference only once after all encoding occurred, our paradigm examined the change in memory inference in face of reoccurring demand, and the age differences therein. Furthermore, we focused on testing children and teenagers with narrower age range than Schlichting et al. [13]. By this, we can more specifically pinpoint the difference between late childhood and adolescence. We hypothesized that children, compared to teenagers and young adults, would show lower memory performance, with a particularly larger age difference for trials that require memory inferences than direct memory associations.

## Method

### Participants

The experiment included three age groups: 25 children (12 female; aged 9–10 years, *M* = 9.44, *SD* = 0.51), 23 teenagers (12 females; aged 12–13 years, *M* = 12.30, *SD* = 0.47), and 20 young adults (11 females, ages 19–25 years, *M* = 23.00, *SD* = 1.95). Participants were recruited from the existing participant database of the Max Planck Institute of Human Development, and were screened for any history of neurological or psychiatric disorders (e.g., ADHD, depression, etc.). Young adults were students of universities in Berlin, and children and teenagers were attending *Gymnasium*, which is the highest academic track in the German schooling system.

Informed written consent was obtained from all subjects or parents of subjects under age 18 before participation in the study, which was approved by the local Ethics Committee of the Max Planck Institute of Human Development and conducted in conformity with the Declaration of Helsinki.

### Material

The stimuli set consisted of 96 images of concrete objects (e.g., fruits, tools, clothes etc.; chosen from [15]) and 64 colour images of human faces (32 females; [16]). The choice of stimuli was made with the consideration that the objects should be familiar to children and teenagers. The selection was validated through two round of screening among co-authors. Objects (A, C) and faces (B) were presented pair wise in pseudo-random and trial-unique combinations, with half the pairs sharing the overlapping face stimuli with the other half. In total, there were 32 AB-pairs and 32 corresponding BC-pairs. As a control, there were also 32 DE pairs that consisted of pseudo-random and trial-unique combinations of faces and objects that did not have overlapping face stimuli.

### Procedure

**Fig 1:**
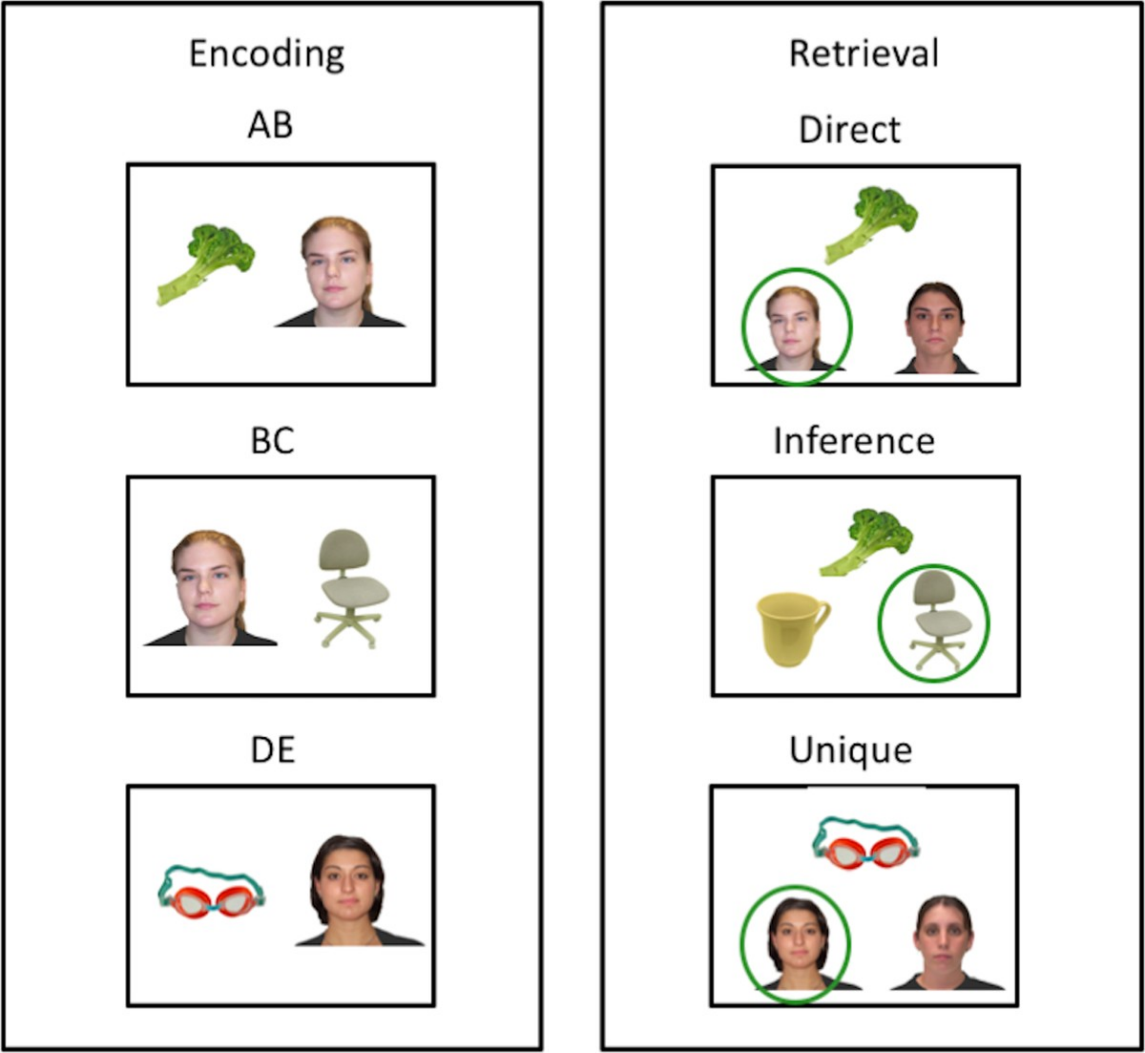
Example stimuli during encoding and retrieval blocks. Note the overlap between AB- with BC-stimuli, and DE-stimuli that are unique during encoding. Stimulus configurations at retrieval consisted of direct (from AB- and BC-stimuli), inference (based on the overlap between AB- and BC-stimuli), and unique trials (from DE-stimuli). Correct choices are circled in green.

We followed the same procedure as Pajkert et al. [14]. The structure of the experiment was as follows (see Fig 1): During encoding blocks, participants were instructed to encode a set of AB- BC-, and DE-associations. During subsequent retrieval blocks, participants were first tested for inferential AC-associations (“inference trials”) and then for memory of direct AB-, BC-, and unique DE- trials. The experiment consisted of a sequence of four cycles, each consisting of an encoding block followed by a delay and a retrieval block.

Encoding blocks consisted of 24 trials (8 AB-, 8 BC-, and 8 DE-trials). Trials were presented in pseudo-random order. AB-trials always preceded the corresponding BC-trials, with a gap of between three and eight trials apart. DE-trials were interleaved with AB- and BC-trials. Within each trial, the stimulus pair was presented for 5 secs followed by a response period. During this period, participants were asked to indicate whether both of the stimuli are living objects or only one (e.g., a plant and a face, a furniture and a face), as a way to maintain participants’ attention on the task. The inter-trial interval varied between 1 and 3 secs.

After the encoding block, participants were given a 5 minutes break, during which the experimenter had a chat with the participant. Retrieval blocks consisted of 32 trials (8 AC-, then 8 AB-, 8 BC-, and 8 DE-trials intermixed). AC-trials were tested at the beginning of the block to prevent re-learning of AB- or BC-associations before testing of AC-associations. In each AC-trial, an A-stimulus was presented in the center of the top half of the screen. Two C-stimuli as choices were presented in the lower half of the screen. Participants were asked to indicate by button press which of the two choices shared an indirect association with the A-stimulus (through a common B-stimulus). A similar structure was followed for the AB-, BC-, and DE-trial. For each trial, either an A-, B-, or D-stimulus was presented in the center of the top half of the screen. Two choice stimuli (either B-, C-, or E-stimuli) were presented in the lower half of the screen and participants were asked to indicate by button press which one of the choices was associated with the top stimulus. In all trials, lure stimuli were always part of other stimuli pairs of the directly preceding encoding block to ensure equality in familiarity between target and lure stimuli. Stimuli were presented until a response was given. All stimuli were only used once in one of the four cycles.

Before the actual task started, participants received instructions about the task with example stimuli, illustrating the nature of direct and indirect associations. Participants also received training of the paradigm using a small set of stimuli (not used in the actual task) that followed the structure of the paradigm. The experimenter checked for participants’ understanding of the task, repeated instruction/training if necessary, before starting the actual task. Therefore, the repeated need to make memory inference decision is made clear to the participants from beginning on.

## Results

Trials in which a response was given within 300 ms were discarded from the analyses as anticipatory response. Only five children, one teenager, and two adults had anticipatory responses (less than three such trials in each one of them). The key measures consisted of percentage of correct response for a given trial type (*unique DE*- *vs*. *inferential AC*-trials) and median of reaction times (RTs) of correct trials of a given trial type. Unique trials are better measures of associative memory compared to the direct trials because of the overlapping structure among the direct trials. Results also remained the same when using direct trials in the analyses. Importantly, for the inference trials, we restricted the calculation to inference trials for which both direct associations (AB, BC) were correctly remembered in the corresponding direct trials. This ensures that a possible inability to make memory inference was not due to having insufficient memory for the direct associations. We first checked for cycle effect, and if there was none, data was collapsed across cycles. The mean number of inference trial (with both direct associations being correct) was 17 for children (range: 9–26 trials), 24 for teenagers (range: 11–32 trials, and 28 for young adults (range: 19–32 trials). Significance level was set at p < .05. Post hoc analysis was performed when necessary with Bonferroni correction for multiple comparisons.

### Accuracy

**Fig 2:**
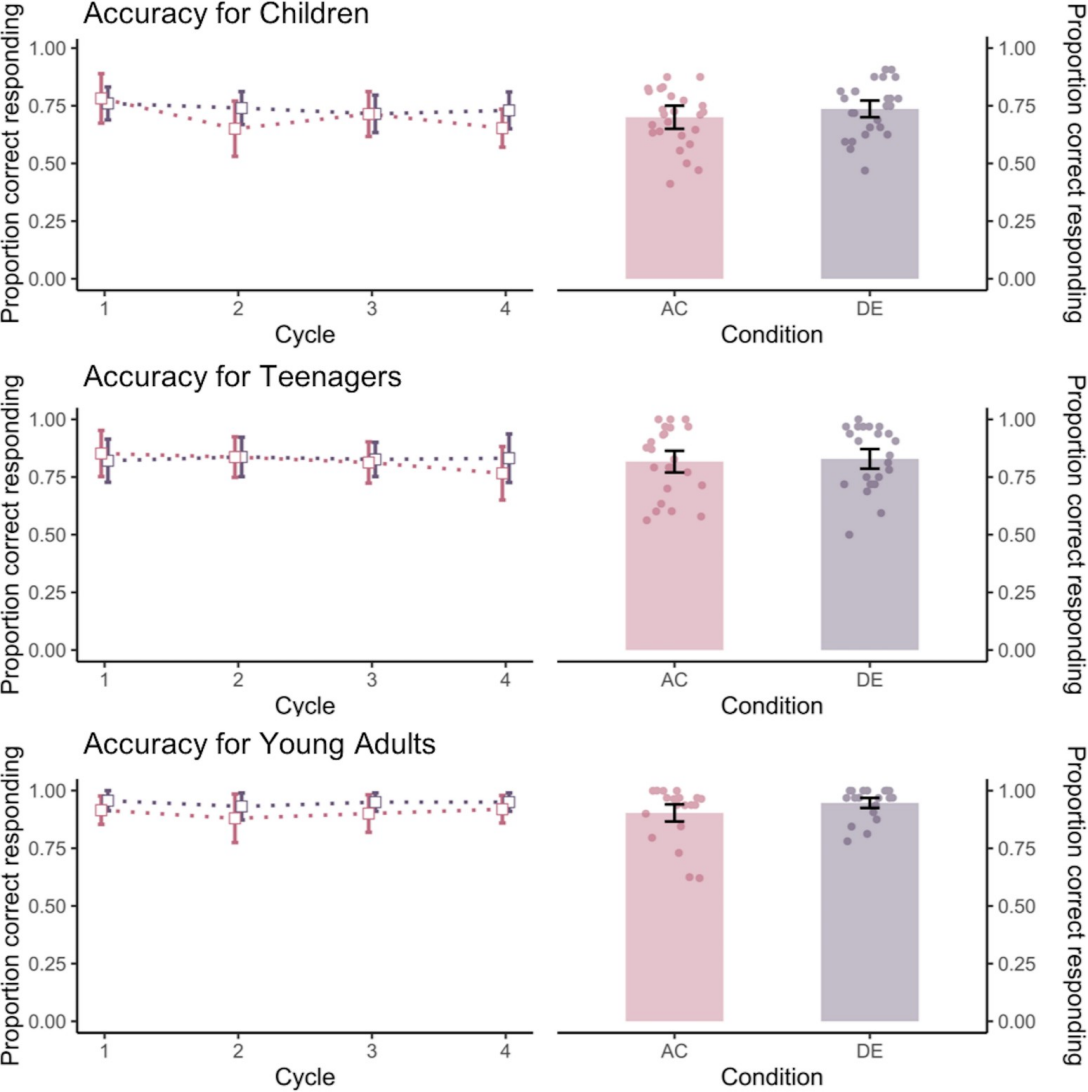
Accuracy of each age group for inference (AC) and unique (DE) trials at each cycle (left) and across all cycles (right).

We first conducted a 4 (cycle) x 2 (trial type) x 3 (age group) mixed ANOVA on the memory accuracy measures. As neither the cycle main effect nor any interaction involving cycle was significant, data was collapsed across cycles. Next, we ran the main 2 (trial types: unique vs. inference) x 3 (age groups: children, teenagers, adults) ANOVA analysis (see data plotted in Fig 2, right panel). There was a significant effect of trial type, *F*(2, 65) = 4.88, *p* = .03, *η2* = .07, showing that the accuracy of unique trials (*M*_*unique*_ = .84, *SD*_*unique*_ = .12) was significantly higher than inference trials (*M*_*inference*_ = .80, *SD*_*inference*_ = .13). There was also a significant effect of age group, *F*(2, 65) = 21.47, *p* < .001, *η2* = .40. Post hoc test showed that children performed significantly lower than adolescents across both trial types (*p* = .003), who performed significantly lower than adults (*p* = .008). However, the interaction between trial type and age group was not significant (*F* < .50, *p* = .62).

### Reaction time

**Fig 3:**
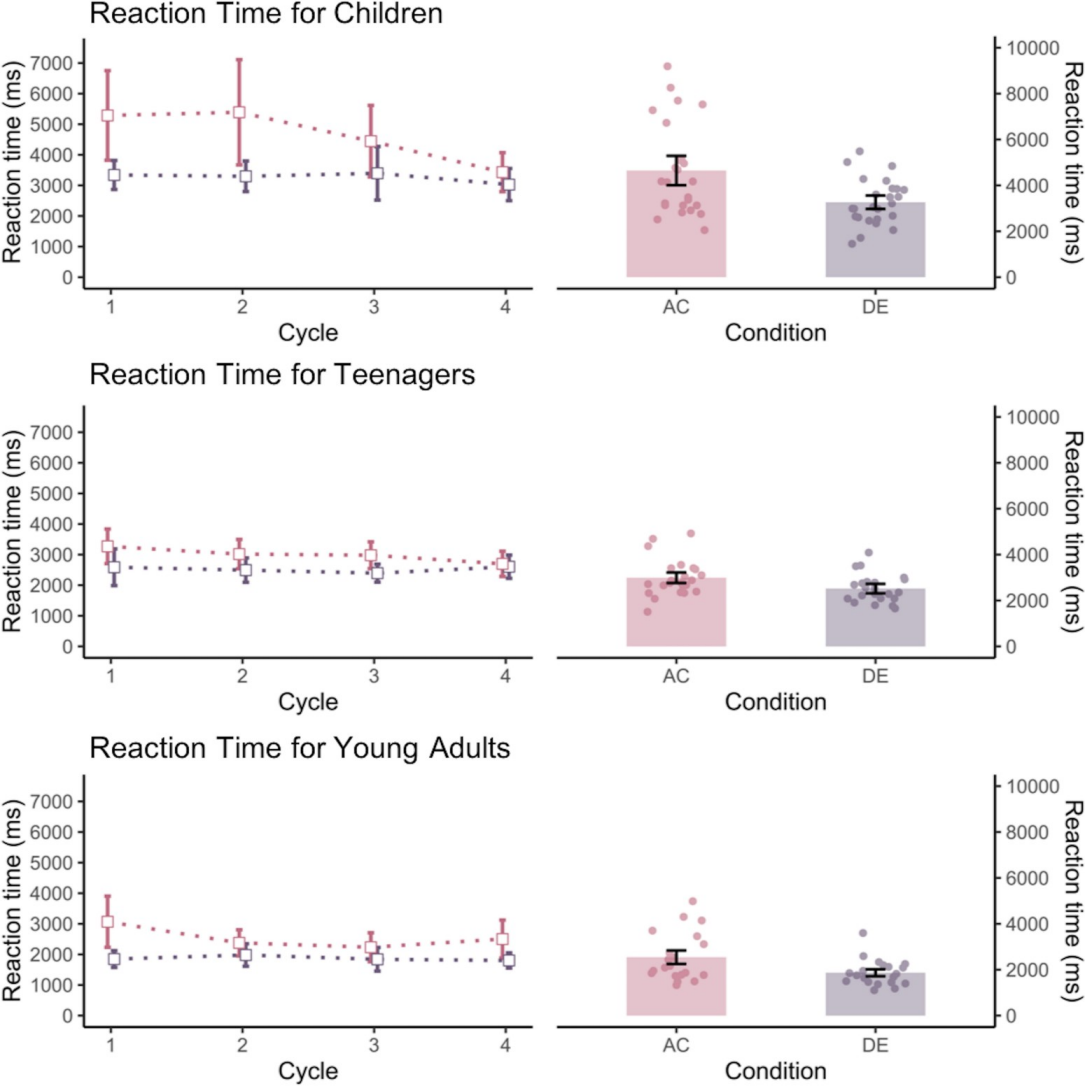
Reaction time of each age group for inference (AC) and unique (DE) trials at each cycle (left) and across all cycles (right).

We first conducted a 4 (cycle) x 2 (trial type) x 3 (age group) mixed ANOVA on the reaction time measures (see data plotted in Fig 3). There was a significant main effect of cycle *F*(3, 56) = 4.68, *p* = .005, *η2* = .20 that is qualified by a cycle by trial type interaction *F*(3, 56) = 2.89, *p* = .04, *η2* = .13. This was driven by a steeper decline in reaction time for the inference trials from cycle 1 (Mean RT = 3920ms) to cycle 4 (Mean RT = 2898ms). In comparison, there was very little decrease in reaction time for the unique trials from cycle 1 (Mean RT = 2514ms) to cycle 4 (Mean RT = 2395ms). There was also a significant trial type effect, *F*(1, 58) = 68.07, *p* < .001, *η2* = .54, a significant age group effect, *F*(2, 58) = 13.80, *p* < .001, *η2* = .32, qualified by a significant interaction between the two, *F*(2, 58) = 8.19, *p* = .001, *η2* = .22. To examine this interaction, we calculated a cost measure by taking the difference in reaction time between the inference and unique trials (across cycles), and then conducted a comparison on this measure. Children showed larger difference in reaction time (Δ = 1387 ms) due to the need for memory integration than teenagers (Δ = 472 ms, *p* = .01) and young adults (Δ = 678 ms, *p* = .07), with the latter two groups being not significantly different from each other (*p* = 1.0). As the age groups differed in the number of trials where both AB and BC trials were correct, we also included actual number of inference trials as a covariate in this analysis, and found that the effect remained similar albeit weakened, F(2, 64) = 2.83, p = .07.

### Correlation between accuracy on direct (AB, BC) and inference (AC) trials

Finally, we explored the correlation between accuracy of direct (AB, BC) and inference trials (AC), controlling for unique trials (DE). We reasoned that forming integrative representation, either at encoding or retrieval, would require memory for the direct associations. Therefore, participants who formed integrative representation should show higher correlation between direct and inference trials, beyond general associative memory ability (DE trials).

We tested for group differences in the partial correlation between AC and AB/BC trials using path models implemented in Mplus. This was done by estimating a multiple group regression model where all variables were standardized within group. AC and AB/BC were regressed on DE trials. The residual correlations between AC and AB/BC of the three age groups were then compared, sequentially in pairs, using likelihood ratio test of models where the correlations were either freely estimated or set to be equal to each other. In a fully saturated (free) model, the estimated partial AC-AB/BC correlation for children was 0.10, for teenagers 0.40, and for young adults 0.75. First, to test for children vs. young adults, the correlation of AC-AB/BC between children and young adults was constrained to be the same, which led to a significant loss of fit, Δχ^2^ = 8.52 (critical value = 3.84), Δdf = 1. This shows that the AC-AB/BC correlation was higher in young adults than in children. At the next step, the correlation of AC-AB/BC between teenagers and young adults was constrained to be the same, which led to a nonsignificant loss of fit, Δχ^2^ = 3.02, Δdf = 1. This shows that the AC-AB/BC correlations of teenagers and young adults were not significantly different from each other. Finally, the correlation of AC-AB/BC between children and teenagers was constrained to be the same, which led to a nonsignificant loss of fit, Δχ^2^ = 1.24, Δdf = 1. This shows that the AC-AB/BC correlations of children and teenagers were not significantly different from each other.

## Discussion

In this study, we compared the accuracy and reaction time of children, teenagers, and young adults on tests of memory-based inferences and memory for associations. Our results showed that young adults outperformed teenagers, who in turn outperformed children in accuracies of both trial types. This was not in line with our hypothesis that predicted a task by age interaction, in which children would perform particularly worse on the inference trials. However, in the reaction time measure, we did find that children were particularly slower than teenagers and young adults in inference trials than in association (unique) trials. In the following, we will elaborate on the implications of these results.

Our results on memory accuracy did not replicate the finding of Schlichting and colleagues [13], which showed that children aged 6 to 11 years of age, compared to teenagers aged 12 to 17 years of age and young adults above 18 years of age, were particularly worse in memory inference than in associative memory performance. Our results rather suggest that the relative accuracy difference between memory association and memory inference was not significantly different across the age groups. There are several divergences between the two studies that warrant attention. First, our children and teenager age ranges (9–10 and 12–13 years) were more restricted than those in [13], in accordance with our intention to more clearly pin point the age gradient of memory inference ability. We expected a particularly large improvement in memory inference that might take place between these two age periods during development, which is, however, not supported by our results. This suggests that a transition in (or onset of) memory inference may happen earlier in development before nine years of age, calling for future studies to include younger age range in a continuous manner. Interestingly, a recent review by Keresztes and colleagues [17] postulated that during development, generalization (such as recognizing regularities across episodes) may be prioritized over remembering specific episodes. This may be supported by pattern completion in the hippocampus, in which incomplete representations are filled-in based on previously stored representations [18]. To the extent that memory inference is related to generalization and that it relies partly on pattern completion, it is plausible that memory inference is earlier developing than initially conceptualized (see [19] for evidence of bias towards pattern completion early in development).

Second, in Schlichting et al. [13], abstract objects were used as stimuli, while in contrast we used concrete objects and faces. Moreover, participants in [13] saw each direct pair four times, potentially to boost their memory for direct associations, and then followed by an inference test of 30 pairs at the end of the experiment. In our case, participants were tested on 8 memory inference trials repeatedly, always with different pairs. Therefore, it is possible that our participants could better prepare for the re-occurring inference tests, leading to less age differences than would be otherwise observable.

Despite not finding the expected age pattern on accuracy, there are two aspects in the results pointing for more nuanced age differences that are novel and interesting. First, on reaction time measures, children, compared to teenagers and young adults, needed longer time for making memory inference decisions than associative memory decision. There are two non-mutually exclusive explanations to this finding. First, children may rely more on making inferences during retrieval, hence needed more time to first retrieve the direct associations and then making the inference. The older age groups may have formed integrated representations across the direct associations during encoding, or are more efficient in doing so during retrieval. Second, making memory inference decision may be particularly effortful for children. It is conceivable that multiple processes underlie tasks that measure memory inference, for example cognitive control processes. Neuroimaging studies suggest that memory inference is mediated by medial- and lateral-prefrontal cortex as well as hippocampus (see review in [2]). This finding is corroborated by studies of patients with hippocampal [14] and ventromedial prefrontal cortex lesions [20]. In a study by Zeithamova and Preston [3], it was found that the prefrontal cortex, particularly inferior frontal gyrus, contributes particularly to inferential processing at retrieval, in line with findings that the region has been implicated in non-mnemonic relational reasoning where multiple relationships need to be considered to infer an unknown relationship [21]. As the lateral prefrontal gyrus is protracted in development [22], this may explain our finding that making memory inference decisions at retrieval is particularly effortful for children, even if they do not show an age-related deficiency in accuracy measure. It is important to point out that in our paradigm we did not tap into the spontaneous formation of integrative representation specifically either at encoding or retrieval, but rather the general ability to integrate across memory associations due to the reoccurring demand to make memory inference, which is an aspect that young adults are more efficient in carrying out than children.

Finally, our exploratory correlation analysis showed that across study cycles, young adults showed higher correlation between accuracy on direct memory association trials and inference trials than children, above and beyond general associative memory ability. The pattern in young adults suggests that they rely closely on AB- and BC-associations, possibly to form integrated ABC-triplet either at encoding or retrieval, for making the inference decisions. Children did not show a clear pattern of correlation across trial types, suggesting that they may be less consistent in making encoding-based integration or retrieval-based inference as young adults do.

There are several open questions that are important to follow up in future investigations. First, the discrepancy of age-related difference on accuracy between our study and the study of Schlichting et al. [13] suggests that task feature, such as whether direct associations are studied several times or demand for inference decision is repeated, can influence age differences in memory inference. A similar discussion existed in the investigation of inference reasoning about the task-situational conditions that influence children’s ability to solve transitivity problems [23]. It would be helpful to carefully manipulate important task features in a within-person design to tease apart the effects of these features. Second, while we postulated that young adults could increasingly form integrated representations of ABC-triplets, it is not clear in our current data whether they formed these representations during the encoding of BC-trials (by recalling the corresponding AB-pairs and forming the triplet representation instantaneously) or at retrieval when inference decision needs to be made. Based on fMRI, these processes can be distinguished by showing differential neural correlates [24]. Another potential route that is more accessible but remains to be demonstrated is to use eye tracking to examine traces of retrieving AB during BC encoding. Hannula and Ranganath [25] showed that relational memory can be evident in eye movement patterns, which is predicted by hippocampal activation even when overt behavioral reports are incorrect. Capitalizing on such characteristics of eye movement and by using an experimental setup that allows for integrative encoding to emerge (e.g., across spatial locations on presentation screen), there may be an alternative way to assess specific processes that are involved in memory integration. Finally, as mentioned before, future studies would benefit from including younger participants, while keeping the age range narrow or utilizing age continuous analyses, in order to fully characterize the age gradient of memory inference development. This would also call for a more extended evaluation of the experimental stimuli and procedure for the young age groups. Currently, it is possible that the encoding task of living/nonliving judgment as well as participants’ different knowledge level of the stimuli may have impacted children’s memory performance.

Taken together, our findings suggest that there may be an age-related shift in how information is integrated across experienced episodes, namely from predominantly making inferences at retrieval during middle childhood to forming integrated representations (at encoding and/or retrieval) in young adulthood.
